# Effect of Dental Glass Fiber Posts on Root Stresses and Fracture Behavior of Endodontically Treated Maxillary Central Incisors: A Finite Element Analysis Study

**DOI:** 10.7759/cureus.43056

**Published:** 2023-08-06

**Authors:** Ahmed A Madfa

**Affiliations:** 1 Department of Restorative Dental Science, College of Dentistry, University of Ha'il, Ha'il, SAU

**Keywords:** tensile stress, restoration of endodontically treated tooth, fracture resistance, post design, glass fiber post

## Abstract

Objective: In this work, the influence of glass fiber posts with different designs on the root stress that had endodontic treatment was examined using the finite element method.

Method: Using two distinct materials (metal and glass fiber) and two different prototypes (tapered and parallel-sided), four three-dimensional (3D) finite element models of an upper central incisor were made and studied. Each 3D model received an oblique loading of 100 N. All forces were dispatched as distributed pressure to the aforementioned region. There were no considerations made for potential stresses when performing the endodontic procedure. The endodontic treatment was conducted without taking into account any potential stressors. The root stresses were then recorded.

Results: The largest tensile stress is often focused at the apical third of the post and post/cement contact, as well as at the coronal third of the root on both the labial and palatal sides of the root, independent of the post's design and material. Restoration of endodontically treated maxillary central incisors with glass fiber posts has been shown to have less stress concentration than titanium posts. Regardless of the post materials employed, the tapered post design generated a higher tensile stress distribution than the parallel side design.

Conclusions: Prefabricated fiber posts used in model restoration resulted in more evenly distributed stress and less concentrated stress on the root. Reduction in modulus of elasticity of post materials used generally shows less stress concentration.

## Introduction

Healthy teeth are less likely than diseased dental canals that have undergone endodontic therapy to fail biomechanically [1]. Teeth have been affected significantly as a result of access cavity and endodontic preparations, which resulted in considerable tissue loss, moisture loss, reduced resistance, and flexibility loss [2,3]. Due to significant tissue loss, decreased moisture content and flexibility, decreased resistance brought on by endodontic access preparations, and increased brittleness, endodontically treated teeth are more likely to shatter than untreated teeth [2,4]. Despite advancements in restorative materials and methods, endodontically treated teeth still experience root fractures [5]. Preserving tooth structure during restorative and endodontic operations is the greatest method for preventing root fractures in teeth that have had endodontic treatment [6]. To properly restore teeth that have received endodontic therapy, better restorative procedures are necessary [6].

Biomechanical failure in teeth that have had endodontic treatment is caused by caries; endodontic access; and changes in physical, chemical, and mechanical characteristics [1,2,7]. Therefore, using a post during root rehabilitation may be necessary [8]. Prefabricated metal and non-metal posts with resin cores have taken the role of custom-made posts and cores. One of the most often used techniques is the prefabricated post and core system, which needs less chair time [9]. However, the two most frequent issues with the prefabricated post and core approach are root fracture and post and core loosening. Root fractures are frequent in clinical practice, according to long-term studies of the post and core approach that have revealed a wide range of survival rates [10-12]. However, numerous researchers discovered that glass fiber posts are a good substitute for metal posts because they have characteristics similar to root dentin [12,13].

Strength tests are a great way to learn more about how stresses and strains affect tooth tissue and the area around it. It is challenging to analyze stress distributions in vivo. As a result, testing techniques are being employed more frequently to gauge the strength of mechanical structures [14,15]. In this list, finite element analysis (FEA) is one. Its core concept is the discretization of the object under study and the substitution of its geometry with a constrained number of appropriate spatial components while maintaining the mechanical and elastic characteristics of the underlying structures. High precision repeatability under constant conditions is made possible by FEA and is based on several factors, including element size and numerical object conformance to the real one [14].

Determining stress distributions in teeth repaired with dental posts after endodontic treatment is challenging due to the post's tiny size and complicated construction. On the other side, there has been debate on the research of stress distribution in teeth that have had endodontic treatment and have dental posts. As a result, this area was chosen as the study's theme. The objective of this FEA study was to examine and compare the impact of tapered versus parallel-sided post designs, using both glass fiber and metal post materials, on resulting stress distributions in the root dentin of endodontically treated maxillary central incisors.

## Materials and methods

**Figure 1:**
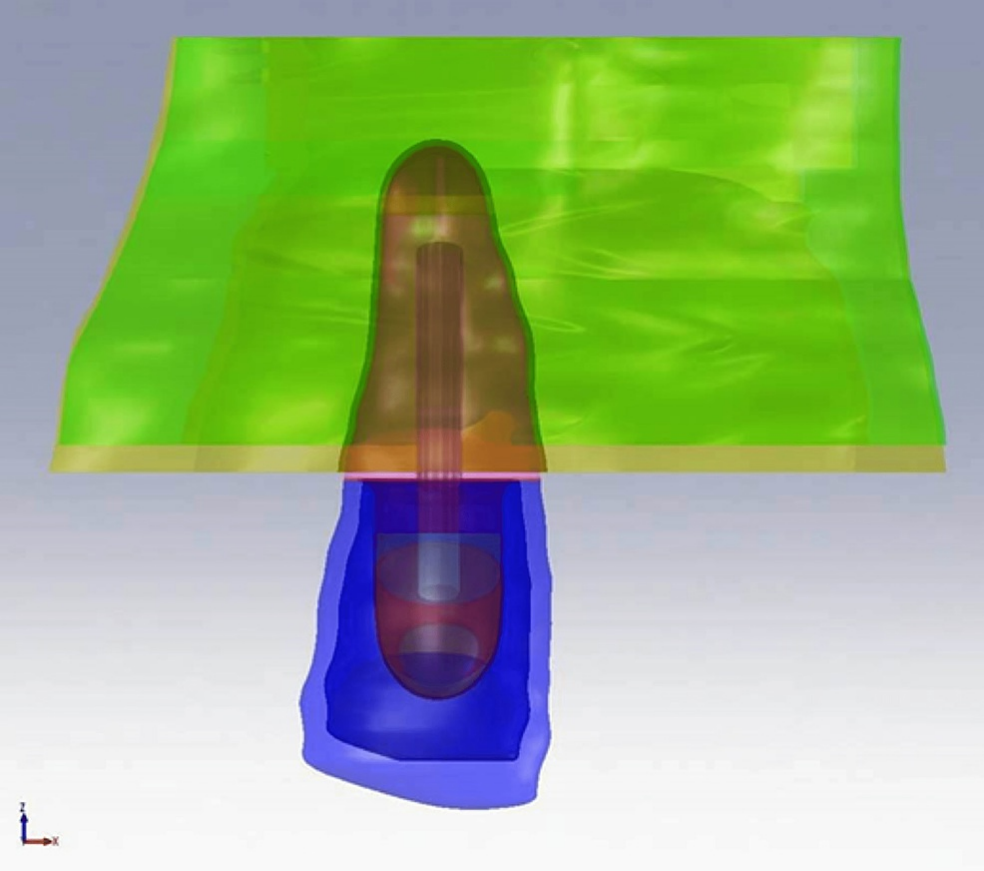
Schematic illustration of the 3D geometric model 3D, three-dimensional

Using computed tomography (CT) data consisting of 98 slices with a thickness of 1 mm for each slice is used to produce a three-dimensional (3D) model of the upper central incisor. A maxillary central incisor and the cortical and cancellous bones that surround it were created in 3D using Mimics (Materialise NV, Belgium) and the Hounsfield unit. The periodontal ligament has a thin texture and a pixel size of 0.4235 mm, making it challenging to define from CT scan images. A periodontal ligament with a thickness of 0.18 mm was developed on the isocurves of the dentine and its volume was deducted from the cortical and cancellous bone's volume. Solid Works (Dassault Systèmes, USA) was used in model restoration methods for teeth that had undergone endodontic treatment, such as dental posts, composite cores, and dental metal-ceramic crowns, depending on the geometry of the root. The dimensions of each item were estimated using information from the literature [10,11,16-18]. A diagram illustration of the geometric model is shown in Figure 1.

In this work, first-order four-node linear tetrahedral solid elements (C3D4) were employed for stress analysis. The continuous stress on the tetrahedral parts of these C3D4s showed limited convergence; therefore, a small mesh was utilized in this study to achieve correct findings. It was assumed that every component would be flawlessly connected, with no spaces between them.

As instructed by the system, the boundary conditions for the nodes were positioned at the bottom end line of the models and fastened to the central incisor's supporting structure. To each 3D model, a 100 N oblique static loading was applied. All forces were dispatched as distributed pressure to the aforementioned region. Any stress that might have been generated during the endodontic procedure was not taken into consideration.

The research was carried out using four different 3D fine element (FE) models of an upper central incisor, two different types of prototypes (tapered and parallel-sided), and two different types of materials (glass fiber and titanium). For each component of the FE model, the mechanical properties of the restorative materials were produced. Each component of the model was created using materials that were believed to behave linearly. Table 1 and Table 2 describe the elastic characteristics of the restorative materials employed in the geometric model.

**Table 1:** Mechanical properties of isotropic materials

Materials	Modulus of elasticity (GPa)	Poisson’s ratio
Dentine	18.60	0.32
Periodontal ligament	0.000069	0.45
Compact bone	13.70	0.30
Cancellous bone	1.37	0.30
Gutta-percha	0.14	0.45
Ceramic crown	69.0	0.28
Metal coping	96.0	0.35
Titanium post	116.0	0.33
Zinc-oxide phosphate	22.0	0.35
Adhesive cement resin	18.6	0.28
Composite resin	12.0	0.33

**Table 2:** Mechanical properties of orthotropic materials

Materials	Glass fiber post
Ex (MPa)	37.0
Ey (MPa)	9.5
Ez (MPa)	9.5
Vxy	0.27
Vxz	0.34
Vyz	0.27
Gxy	3100
Gxz	3500
Gyz	3100

Tensile stress for glass fiber posts with different designs at three planes (xy, xz, and yz) was analyzed using the Solid Works software (Dassault Systèmes, USA). The concentration of tensile stress at posts central and interfacial of post/cement-dentine was examined.

## Results

**Figure 2:**
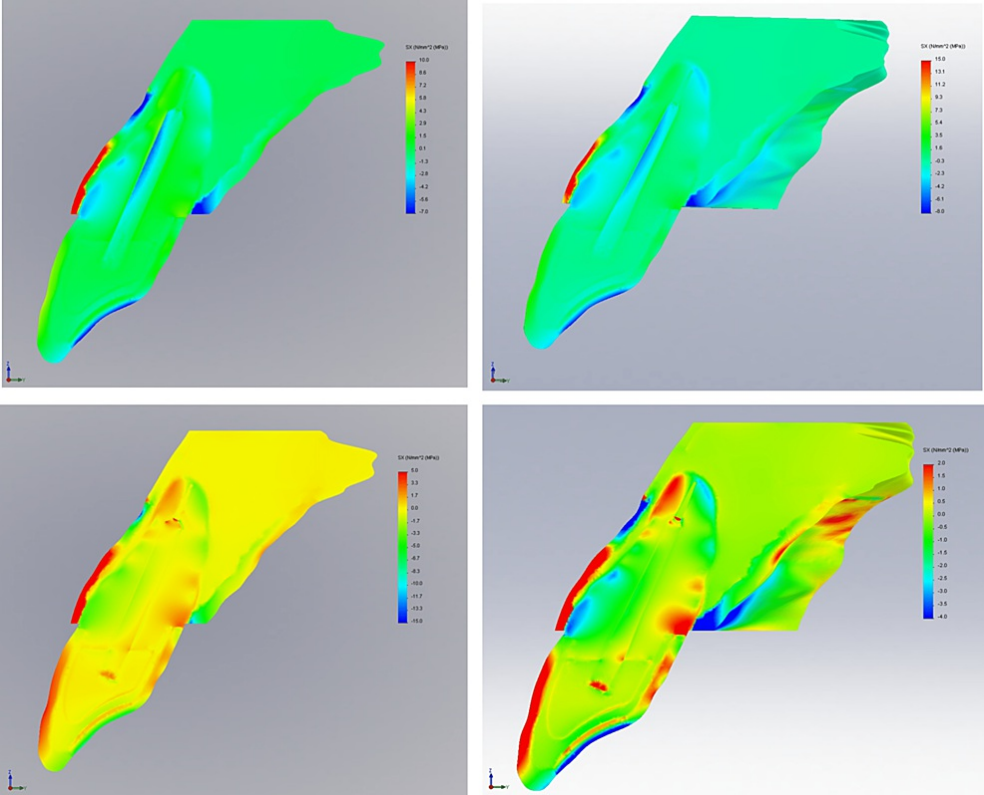
Contour plots of the stress distribution following oblique loading in the XY direction

**Figure 3:**
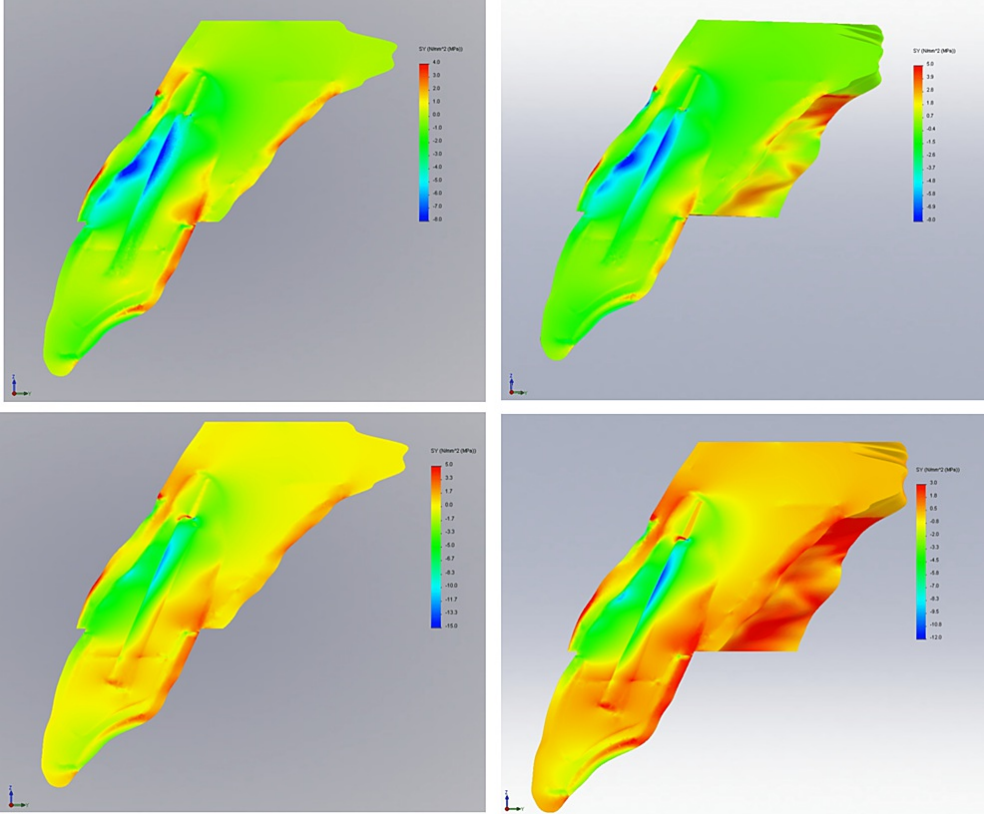
Contour plots of the stress distribution following oblique loading in the XZ direction

**Figure 4:**
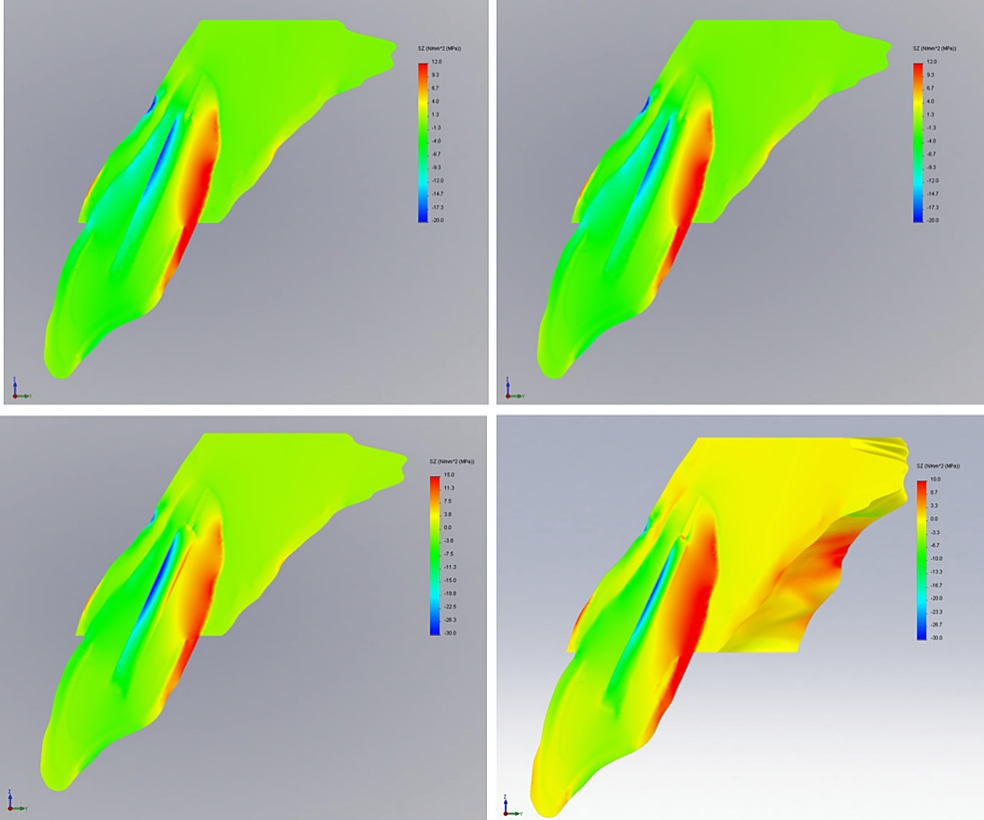
Contour plots of the stress distribution following oblique loading in the ZY direction

Tensile stress for four different 3D FE models of an upper central incisor restored with two different types of prototypes (tapered and parallel-sided) and two different types of materials (glass fiber and titanium) at three various planes (xy, xz, and yz) following oblique loading of 100 N are displayed in Figure 2, Figure 3, and Figure 4.

The largest tensile stress is often focused at the apical third of the post and cement/dentine contacts, as well as at the coronal third of the root on both the labial and palatal sides of the root, regardless of the post's design and material. Glass fiber dental posts have shown to have less stress concentration than titanium posts. From the coronal to the apical regions of the post and dentine interaction, the glass fiber post efficiently absorbed interface stress.

The largest tensile stress, however, was experienced by tapered titanium post (41.902 MPa), followed by parallel titanium post (37.554 MPa) as shown in Table 3. The tapered post design generated a larger tensile stress distribution than the parallel side design, regardless of the post materials used.

**Table 3:** Maximum stress distribution following oblique loading

	Glass fiber post		Titanium post	
	Tapered	Parallel	Tapered	Parallel
	Max stress (MPa)	Max stress (MPa)	Max stress (MPa)	Max stress (MPa)
Dentin	19.208	18.166	21.125	20.723
Cancellous bone	48.588	46.09	48.596	45.673
Cortical bone	67.638	64.005	67.68	63.9
Post	21.374	17.863	41.902	37.554
Post cement	16.547	13.994	28.933	26.678
Post-cement interface	16.547	13.994	28.933	26.578
Crown cement	22.809	22.142	22.163	21.845
Core	6.0034	5.6553	9.9503	10.004
Metal coping	12.349	10.994	12.177	10.921
Porcelain	22.809	22.142	22.163	21.845

The maximum interfacial tensile stress values between dental post and cement are shown in Table 3. It was noticed that the parallel glass fiber post generated a lower tensile stress (13.994 MPa), which was then tapered once (16.547 MPa). However, the maximum interfacial tensile stress concentration was perceived model restored by tapered titanium (28.933 MPa) followed by parallel titanium posts (26.578 MPa).

## Discussion

Endodontically treated teeth weaken and are more likely to deteriorate as a result of the significant loss of tooth structure. Owing to the significant loss of tooth structure during root canal treatment, teeth become weaker and more vulnerable to break.

Endodontically treated teeth nevertheless experience problems with restoration because they are more prone to experience biomechanical failure because of severe tooth loss, coronal and/or root cavities, extensive restoration, occlusal imbalance, and intracanal retainer preparation [1]. The tooth must be supported by intracanal structures.

Numerous methods, including photoelasticity, FEA, strain gauges, holography, and other computational approaches, have been used to investigate stresses in tooth structures. The FEA may be carried out to study the interactions between different designs and the biomechanical system. FEA can only offer a rough simulation of the tooth because it is a complex living tissue. Stress is produced in the dentin and the tissues around the tooth as a result of chewing and biting forces being transmitted to the post. A post that is under too much tension could fracture. Tooth fracture, post-fracture, post-loosening, and other problems could occur as a result of excessive stress. The utilization of finite element methods in stress analysis offers the distinct advantage of ensuring uniformity in studying the mechanical behavior of dental materials under all circumstances. Furthermore, the quantitative method ensures that the size and taper of each model's root canal preparation are identical, which would have been difficult to do in an experimental investigation using human teeth. This strategy, however, just offers an approximation. Therefore, rather than using a quantitative technique, the study's findings are presented in a qualitative manner [16-19].

The strong metallic posts' high elasticity modulus prevented the dentin tissue, which had a less rigid structure, from absorbing stress energy or receiving oblique compressive stresses. A root fracture is more likely under these circumstances. The researchers reported that root fractures in teeth restored using cast posts were influenced by the modulus of elasticity of dental posts [15,20,21]. Pereria et al. used prefabricated metal, carbon fiber, and glass fiber posts and evaluated their fracture resistance [22]. Root fracture was not detected in the other groups, despite catastrophic fracture being detected in every tooth in the metal post group. The metallic post also showed more resistance than the glass fiber post. The fracture resistance of the metallic post (2/3) and the fiber posts (1/3,1/2, and 2/3) that were applied at various lengths were compared by Franco and colleagues, who came to the conclusion that the metallic post had a higher fracture resistance value than the fiber posts [23]. In vitro stress distributions of the metallic and fiber posts were investigated by Verrisimo et al. who found that the glass fiber posts had a more uniform stress distribution whereas the metallic posts put more stress on the post-dentin interface [24]. The metallic posts caused greater stress overall. These findings are consistent with the current investigation, which discovered that titanium posts had a more uneven stress distribution than glass fiber posts. The glass fiber post effectively absorbed interface stress from the coronal to apical areas of the post and dentine interface.

The elastic modulus of the post material affects the amounts of tensile stress in posts. The current analysis found that models incorporating posts made of higher elastic modulus metal materials resulted in increased tensile stress values within the post itself compared to models with lower modulus fiber posts. The post's rigidity affects the tensile strains at the post/dentin contact under oblique load; the higher the post's rigidity, the lower the strains. The cingulum received an oblique load, whereas the incisal received a vertical load. The maximum tensile stress for an oblique load was found in the cervical area and the cingulum. Results indicated that fiber posts resisted forces better than metallic posts and put higher stress on dentin. Therefore, compared to a fiber post, the metallic post has superior fracture resistance.

The two stress concentration zones were found to be the middle and cervical thirds of the root, where the cortical bone terminates on the root, and the cervical region of the root, which was covered by the cervical borders of the crown. In this investigation, compressive pressures were localized on the buccal side at both sites of the models, whereas tensile stresses were focused on the palatal side. The cervical region of the root has been identified as a stress accumulation location in several investigations [25-27].

Among the several post designs, tapered metal posts were determined to be the most hazardous to the fracture resistance of post-restored teeth [28, 29]. This is consistent with the findings of the current study, which showed that tapered posts created more stress than parallel-side posts when the kind of material employed was disregarded. In addition, mounting data shows that tapered posts wedge the root, increasing the likelihood of fracture and predisposing the tooth to loss of retention.

The fact that a metal post resists more stress and absorbs a larger portion of the pressure put on the teeth than a fiber post can be used to summarize this supposition. The maximum stress is visible in the cervical region of the crown when the stress pattern and the location of the stress are taken into account, that is, for an oblique load, the load is applied in the cingulum region. The assumption may be drawn from the fact that the cervical area of the tooth is near the fracture line on both the metal post and the fiber post.

## Conclusions

Within the limitations of this investigation, the glass fiber post used in model restoration resulted in more evenly distributed stress and less concentrated stress. Reduced stiff materials generally show less stress concentration. The utilization of a glass fiber post-core system has been found to result in a greater improvement in fracture resistance for endodontically treated maxillary central incisors compared to titanium post-core systems.

Since restorative materials have such a wide range of elastic modulus, non-uniform stress distribution and accumulation of stresses in different areas may occur, posing a risk to the survival of already damaged teeth and restorations. Such combinations should be avoided, and a post with a modulus elasticity similar to that of dentine should be chosen.

## References

[1] Tang W, Wu Y, Smales RJ (2010). Identifying and reducing risks for potential fractures in endodontically treated teeth. J Endod.

[2] Soares CJ, Santana FR, Silva NR, Preira JC, Pereira CA (2007). Influence of the endodontic treatment on mechanical properties of root dentin. J Endod.

[3] Comba A, Baldi A, Saratti CM (2021). Could different direct restoration techniques affect interfacial gap and fracture resistance of endodontically treated anterior teeth?. Clin Oral Investig.

[4] Jurema AL, Penteado MM, Tribst JP, Caneppele TM, Borges AL (2020). Effect of glass-fiber post on the biomechanical behavior of teeth with direct veneers. Braz Dent Sc.

[5] Eliyas S, Jalili J, Martin N (2015). Restoration of the root canal treated tooth. Br Dent J.

[6] Sathorn C, Palamara JE, Palamara D, Messer HH (2005). Effect of root canal size and external root surface morphology on fracture susceptibility and pattern: a finite element analysis. J Endod.

[7] Carvalho MA, Lazari PC, Gresnigt M, Del Bel Cury AA, Magne P (2018). Current options concerning the endodontically-treated teeth restoration with the adhesive approach. Braz Oral Res.

[8] Theodosopoulou JN, Chochlidakis KM (2009). A systematic review of dowel (post) and core materials and systems. J Prosthodont.

[9] Baraban DJ (1988). The restoration of endodontically treated teeth: an update. J Prosthet Dent.

[10] Nahar R, Mishra SK, Chowdhary R (2020). Evaluation of stress distribution in an endodontically treated tooth restored with four different post systems and two different crowns: a finite element analysis. J Oral Biol Craniofac Res.

[11] Badami V, Ketineni H, Pb S, Akarapu S, Mittapalli SP, Khan A (2022). Comparative evaluation of different post materials on stress distribution in endodontically treated teeth using the finite element analysis method: a systematic review. Cureus.

[12] Naumann M, Koelpin M, Beuer F, Meyer-Lueckel H (2012). 10-year survival evaluation for glass-fiber-supported postendodontic restoration: a prospective observational clinical study. J Endod.

[13] Santos AF, Meira JB, Tanaka CB (2010). Can fiber posts increase root stresses and reduce fracture?. J Dent Res.

[14] Wakabayashi N, Ona M, Suzuki T, Igarashi Y (2008). Nonlinear finite element analyses: advances and challenges in dental applications. J Dent.

[15] Ichim I, Schmidlin PR, Kieser JA, Swain MV (2007). Mechanical evaluation of cervical glass-ionomer restorations: 3D finite element study. J Dent.

[16] Chieruzzi M, Pagano S, Cianetti S, Lombardo G, Kenny JM, Torre L (2017). Effect of fibre posts, bone losses and fibre content on the biomechanical behaviour of endodontically treated teeth: 3D-finite element analysis. Mater Sci Eng C Mater Biol Appl.

[17] Zarow M, Vadini M, Chojnacka-Brozek A (2020). Effect of fiber posts on stress distribution of endodontically treated upper premolars: finite element analysis. Nanomaterials (Basel).

[18] Abduljawad M, Samran A, Kadour J, Karzoun W, Kern M (2017). Effect of fiber posts on the fracture resistance of maxillary central incisors with Class III restorations: an in vitro study. J Prosthet Dent.

[19] Agarwal SK, Mittal R, Singhal R, Hasan S, Chaukiyal K (2020). Stress evaluation of maxillary central incisor restored with different post materials: a finite element analysis. J Clin Adv Dent.

[20] Zhu Z, Dong XY, He S, Pan X, Tang L (2015). Effect of post placement on the restoration of endodontically treated teeth: a systematic review. Int J Prosthodont.

[21] Iaculli F, Rengo C, Lodato V, Patini R, Spagnuolo G, Rengo S (2021). Fracture resistance of endodontically-treated maxillary premolars restored with different type of posts and direct composite reconstructions: a systematic review and meta-analysis of in vitro studies. Dent Mater.

[22] Pereira JR, do Valle AL, Shiratori FK, Ghizoni JS, Bonfante EA (2014). The effect of post material on the characteristic strength of fatigued endodontically treated teeth. J Prosthet Dent.

[23] Franco EB, Lins do Valle A, Pompéia Fraga de Almeida AL, Rubo JH, Pereira JR (2014). Fracture resistance of endodontically treated teeth restored with glass fiber posts of different lengths. J Prosthet Dent.

[24] Veríssimo C, Simamoto Júnior PC, Soares CJ, Noritomi PY, Santos-Filho PC (2014). Effect of the crown, post, and remaining coronal dentin on the biomechanical behavior of endodontically treated maxillary central incisors. J Prosthet Dent.

[25] de Castro Albuquerque R, Polleto LT, Fontana RH, Cimini CA (2003). Stress analysis of an upper central incisor restored with different posts. J Oral Rehabil.

[26] Toksavul S, Zor M, Toman M, Güngör MA, Nergiz I, Artunç C (2006). Analysis of dentinal stress distribution of maxillary central incisors subjected to various post-and-core applications. Oper Dent.

[27] Madfa AA, Yue XG, Senan EM (2017). Tensile stress distribution in maxillary central incisors restored with cast-made and prefabricated dental posts. J Oral Research.

[28] Peutzfeldt A, Sahafi A, Asmussen E (2008). A survey of failed post-retained restorations. Clin Oral Investig.

[29] Al-Omiri MK, Mahmoud AA, Rayyan MR, Abu-Hammad O (2010). Fracture resistance of teeth restored with post-retained restorations: an overview. J Endod.

